# Perioperative Inflammatory Biomarkers and Surgical Site Infection After Pelvic and Acetabular Fracture Surgery: A Retrospective Cohort Study

**DOI:** 10.3390/jcm15186981

**Published:** 2026-09-09

**Authors:** Kemal Şibar, Rohat Genç, Mehmet Utku Çiftçi, Mehmet Nur İneci, Hakan Zeybek, Cengiz Yıldırım

**Affiliations:** Department of Orthopedics and Traumatology, Sultan 2. Abdülhamid Han Training and Research Hospital, Selimiye, Tibbiye Street, Istanbul 34668, Türkiye

**Keywords:** pelvic fracture, acetabular fracture, surgical site infection, systemic inflammation response index, neutrophil-to-lymphocyte ratio

## Abstract

**Background**: Surgical site infection (SSI) remains a serious complication following operative treatment of pelvic and acetabular fractures. Although routinely available inflammatory biomarkers have been investigated for perioperative risk assessment in various orthopedic populations, their association with SSI after pelvic and acetabular fracture surgery remains unclear. This study aimed to evaluate the association between perioperative inflammatory biomarkers and postoperative SSI in patients undergoing surgical fixation of pelvic and acetabular fractures. **Methods**: Adult patients who underwent definitive surgical fixation for pelvic ring and/or acetabular fractures between January 2018 and January 2025 were retrospectively reviewed. Hematological parameters and inflammatory biomarkers, including the neutrophil-to-lymphocyte ratio (NLR), platelet-to-lymphocyte ratio (PLR), systemic immune-inflammation index (SII), and systemic inflammation response index (SIRI), were assessed at admission, preoperatively, and on postoperative day 0. Factors associated with SSI were evaluated using univariable Cox regression and Firth penalized logistic regression, with adjustment for treatment strategy, time to definitive surgery, age, sex, and Injury Severity Score. Exploratory receiver operating characteristic analyses were performed to assess the discriminatory ability of preoperative laboratory parameters and inflammatory biomarkers. **Results**: Among 104 patients, 12 (11.5%) developed SSI. Univariable Cox regression identified staged treatment (*p* = 0.015), longer time from injury to definitive surgery (*p* = 0.023), lower admission platelet count (HR 0.61 per 50 × 10^9^/L increase, 95% CI 0.38–0.97; *p* = 0.038), and higher admission SIRI (HR 1.03, 95% CI 1.00–1.05; *p* = 0.048) as factors associated with SSI. In the multivariable Firth-penalized logistic regression analysis, neither staged treatment nor time to definitive surgery remained statistically significant, and none of the included covariates demonstrated a statistically significant independent association with SSI. Exploratory ROC analyses showed limited discriminatory performance of the evaluated preoperative laboratory parameters and inflammatory biomarkers. **Conclusions**: Routinely available perioperative inflammatory biomarkers demonstrated limited utility for identifying patients at risk of SSI following pelvic and acetabular fracture surgery. The associations of admission SIRI, admission platelet count, staged treatment, and delayed definitive fixation observed in univariable analyses should be considered exploratory. These findings highlight the multifactorial nature of postoperative SSI and warrant validation in larger prospective cohorts.

## 1. Introduction

Pelvic and acetabular fractures are among the most severe injuries encountered in orthopedic trauma surgery. While pelvic fractures demonstrate a bimodal age distribution, occurring after high-energy trauma in younger patients and low-energy falls in the elderly, acetabular fractures remain predominantly associated with high-energy mechanisms and frequently require complex surgical reconstruction [1,2]. These injuries are commonly accompanied by substantial blood loss, multiple associated injuries, prolonged hospitalization, and considerable functional impairment, making their management particularly demanding [3]. Despite continuous advances in trauma systems, perioperative care, and surgical techniques, operative treatment of pelvic and acetabular fractures continues to be associated with a substantial risk of postoperative complications that may adversely affect both clinical outcomes and healthcare utilization [4].

Among postoperative complications, surgical site infection (SSI) remains one of the most devastating because it may compromise fracture healing, prolong hospitalization, increase healthcare costs, and frequently necessitate repeated surgical debridement, prolonged antibiotic therapy, or implant removal [5]. Reported SSI rates following operative treatment of acetabular and pelvic fractures vary across the literature and remain a clinically important concern because of their associated morbidity and need for additional surgical procedures [6,7,8]. Previous studies have identified several patient- and surgery-related risk factors for SSI, including obesity, prolonged operative duration, severe associated injuries, Morel-Lavallée lesions, intensive care unit admission, and preoperative inflammatory status [4,7,8]. Although these factors may help identify patients at increased risk, accurately predicting which individuals will ultimately develop postoperative SSI remains challenging in daily clinical practice.

The search for readily available biomarkers capable of improving perioperative risk stratification has therefore attracted increasing attention in orthopedic surgery. Composite inflammatory indices derived from routine blood tests are thought to better reflect the interplay between inflammation and host immune response than individual hematologic parameters alone [9,10]. Several studies have demonstrated significant associations between inflammatory biomarkers and a wide range of postoperative complications, including infectious complications, medical complications, and mortality [11,12,13]. Nevertheless, the predictive performance of individual biomarkers has been inconsistent across different orthopedic populations, and their clinical utility in patients undergoing surgery for pelvic and acetabular fractures remains largely unexplored.

Despite these promising findings, most studies investigating inflammatory biomarkers have been conducted in geriatric hip fracture patients, joint arthroplasty, or spine surgery, where patient characteristics, injury mechanisms, and postoperative courses differ substantially from those of pelvic and acetabular fractures [9,12,13,14]. Patients with pelvic and acetabular fractures often sustain high-energy trauma, experience significant soft-tissue injury and hemorrhage, and frequently require staged surgical treatment or prolonged intensive care, all of which may influence the systemic inflammatory response [3,8]. Consequently, the clinical utility of these readily available biomarkers in patients undergoing surgery for pelvic and acetabular fractures remains unclear. Against this background, we aimed to investigate the association between inflammatory biomarkers and postoperative surgical site infection in adult patients undergoing surgical treatment for pelvic and acetabular fractures.

## 2. Materials and Methods

This retrospective cohort study was conducted at the Department of Orthopedics and Traumatology of a tertiary trauma center. The study protocol was approved by the Sultan 2. Abdülhamid Han Training and Research Hospital Clinical Research Ethics Committee (Application No: 2026-13, Approval No: 8; 25 February 2026) and was performed in accordance with the principles of the Declaration of Helsinki. Because of the retrospective study design, the requirement for informed consent was waived by the ethics committee.

**Figure 1:**
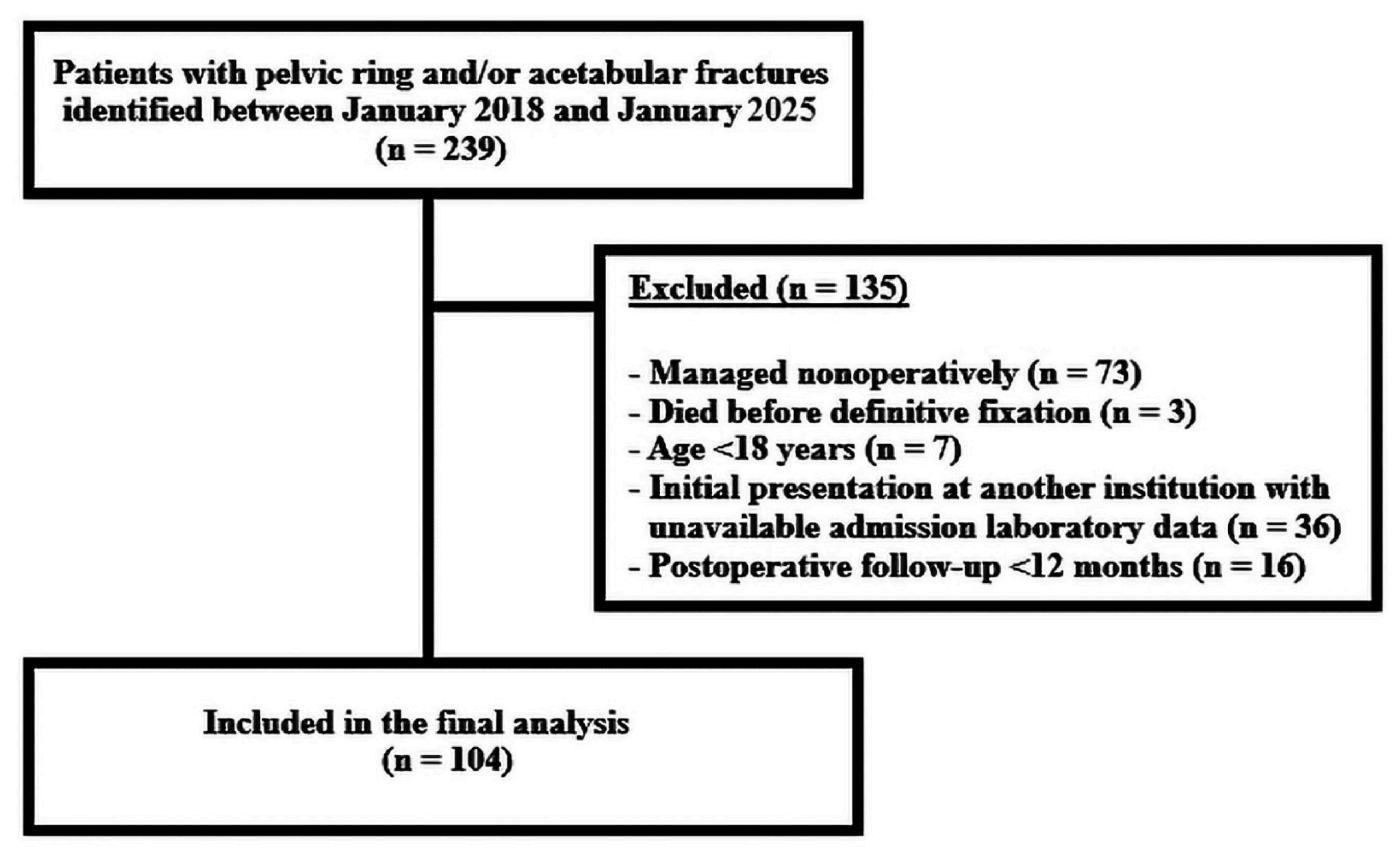
Flow diagram of patient selection for the study.

Adult patients who underwent treatment for pelvic ring and/or acetabular fractures between January 2018 and January 2025 were screened for eligibility. Patients younger than 18 years of age, those managed nonoperatively, patients who died before definitive surgical fixation, patients whose initial presentation was at another institution resulting in unavailable admission laboratory data, and those with less than 12 months of postoperative follow-up were excluded. Consequently, only patients who underwent definitive surgical fixation, had available admission and complete perioperative laboratory data, and completed at least 12 months of follow-up were included in the final analysis. All consecutive patients meeting the eligibility criteria during the study period were included (Figure 1). Because of the retrospective study design, no a priori sample size calculation was performed.

### 2.1. Clinical and Perioperative Variables

The following demographic and perioperative variables were recorded: age, sex, diabetes mellitus, fracture location (pelvic ring or acetabular fracture), treatment strategy (direct definitive fixation or staged management with temporary external fixation), interval between injury and definitive surgery, operative duration, and total and postoperative hospital length of stay. Additional baseline variables including comorbidities, injury mechanism, associated injuries, surgical approach, drain use, anticoagulant use, and intensive care unit admission were also recorded and compared between groups. Injury severity was quantified using the Injury Severity Score (ISS), which was retrospectively determined from the documented injuries in the medical records. The presence of open fractures and Morel-Lavallée lesions was additionally assessed from the medical records. All patients received standardized perioperative antibiotic prophylaxis consisting of 2 g of intravenous cefazolin administered within 30 min before skin incision, followed by 1 g every 8 h for a total prophylaxis duration of 24 h.

### 2.2. Laboratory Assessment

Laboratory measurements were obtained at three predefined perioperative time points: admission (the first laboratory assessment after presentation to the emergency department), the preoperative period (the routine preoperative laboratory assessment obtained within 24 h before definitive fixation), and postoperative day 0 (the first laboratory assessment after definitive fixation). Hemoglobin level, neutrophil count, monocyte count, lymphocyte count, and platelet count were recorded at each time point. Inflammatory biomarkers were calculated using these laboratory parameters, including the neutrophil-to-lymphocyte ratio (NLR), platelet-to-lymphocyte ratio (PLR), systemic immune-inflammation index (SII), and systemic inflammation response index (SIRI). NLR was calculated as neutrophil count/lymphocyte count, PLR as platelet count/lymphocyte count, SII as platelet count × neutrophil count/lymphocyte count, and SIRI as neutrophil count × monocyte count/lymphocyte count.

### 2.3. Outcome Assessment

The primary outcome of the study was postoperative surgical site infection. Patients were categorized according to the presence or absence of SSI during follow-up. The diagnosis of SSI was identified from the institutional medical records and classified as superficial or deep according to the Centers for Disease Control and Prevention (CDC) criteria [15]. All patients had a minimum postoperative follow-up of 12 months. For patients who developed SSI, the interval between definitive surgery and infection diagnosis was also recorded.

### 2.4. Statistical Analysis

All statistical analyses were performed using R statistical software (version 4.5.2). Categorical variables are presented as numbers and percentages [*n* (%)], whereas continuous variables are summarized as mean ± standard deviation, median (first–third quartile; Q1–Q3), and minimum and maximum values. The normality of continuous variables was assessed using the Shapiro–Wilk test. Categorical variables were compared using Pearson’s chi-square test or Fisher’s exact test. Continuous variables were compared between groups using the Wilcoxon rank-sum (Mann–Whitney U) test. Univariable Cox regression models were constructed to investigate factors associated with SSI.

Given the limited number of SSI events (*n* = 12), a single prespecified multivariable model was fitted using Firth-penalized-likelihood logistic regression to reduce small-sample bias and the risk of unstable estimates associated with conventional maximum-likelihood logistic regression. The model included definitive treatment strategy (staged versus direct), time from injury to definitive surgery (per day), age (per 10-year increase), sex, and Injury Severity Score (ISS; per 5-point increase). Confidence intervals were obtained from the profile penalized likelihood. No variable selection was performed. Adjusted odds ratios (ORs) with 95% confidence intervals (CIs) were reported.

Exploratory receiver operating characteristic (ROC) curve analyses were performed for the preoperative laboratory parameters and inflammatory biomarkers evaluated in the study to assess their ability to discriminate between patients with and without SSI. Areas under the curve (AUCs) with 95% confidence intervals (CIs) and two-sided tests against the null value of 0.50 were calculated using the DeLong method. Exploratory cutoff values were determined using the maximum Youden index, with corresponding sensitivity and specificity estimates. Given the limited number of SSI events and the data-driven selection of thresholds, these analyses were considered exploratory, and the identified cutoffs were not interpreted as validated clinical thresholds.

Temporal trajectories of each laboratory parameter were analyzed using linear mixed-effects models. For each parameter, the outcome was modeled as a function of time (three levels: admission, preoperative measurement within 24 h before definitive fixation, and postoperative day 0; reference = admission), SSI status (reference = no SSI), and the time × SSI interaction, with a patient-level random intercept accounting for within-subject correlation. Models were estimated using maximum likelihood, and denominator degrees of freedom for fixed effects were obtained using the Satterthwaite approximation. Fixed effects were evaluated using Type III analysis of variance. Parameters with a pooled sample skewness greater than 1 were natural-log transformed before analysis, with a constant offset applied when the minimum observed value was not positive. Transformation decisions were based on the marginal distribution and were therefore independent of time and SSI status. No missing values were observed. The prespecified primary test for each parameter was the omnibus Type III time × SSI interaction. The resulting *p* values constituted the multiple-comparison family and were adjusted using the Benjamini–Hochberg procedure with a false discovery rate of 0.05. Where the interaction term was estimable, estimated marginal means and pairwise contrasts were derived for SSI groups within each time point and for time points within each SSI group, with Holm adjustment applied within each parameter. Model adequacy was assessed using quantile–quantile and residual-versus-fitted plots, Shapiro–Wilk tests of residuals, and checks for singular fits.

Given the exploratory nature of the study and the number of laboratory parameters evaluated, the potential for type I error due to multiple testing was considered when interpreting the results. Multiplicity adjustments were applied where appropriate for sets of related comparisons, whereas univariable between-group and Cox regression analyses were interpreted as exploratory and hypothesis-generating. All statistical tests were two-sided, and a *p* value < 0.05 was considered statistically significant.

## 3. Results

The study cohort consisted of 104 patients who underwent operative treatment for pelvic or acetabular fractures. The median age was 41 years (interquartile range [IQR], 28–53), and 75 patients (72.1%) were male. Acetabular fractures accounted for 63 cases (60.6%), whereas 41 patients (39.4%) sustained pelvic ring injuries. Definitive fixation was performed primarily in 88 patients (84.6%), whereas 16 patients (15.4%) underwent staged treatment with temporary external fixation before definitive surgery. The median interval between injury and definitive surgery was 5.0 (IQR, 3.0–6.5) days, and the median operative duration was 180 (IQR, 120–240) minutes. No patients in the study cohort had an open fracture or a documented Morel-Lavallée lesion. All patients received the standardized perioperative antibiotic prophylaxis protocol.

During follow-up, surgical site infection (SSI) developed in 12 patients (11.5%). Four infections (3.8%) were classified as superficial and eight (7.7%) as deep. The median time to SSI diagnosis was 16.0 (IQR, 14.5–22.5) days after surgery. Patients were followed for a median of 45 months (IQR, 25–55).

Age, sex, diabetes mellitus, hypertension, coronary artery disease, chronic obstructive pulmonary disease, chronic kidney disease, anticoagulant use, injury mechanism, fracture location, associated injuries, definitive surgical approach, drain use, and preoperative or postoperative intensive care unit admission were comparable between patients who developed SSI and those who did not (all *p* > 0.05). Injury Severity Score was comparable between patients with and without SSI (median, 4.0 [IQR, 4.0–9.0] vs. 4.0 [IQR, 0.0–9.0], respectively; *p* = 0.883).

Staged treatment was more frequently required in the SSI group than in the non-SSI group (41.7% vs. 12.0%, *p* = 0.019). The interval between injury and definitive surgery was longer in the SSI group (median, 6.5 [IQR, 4.5–10.0] vs. 5.0 [IQR, 3.0–6.0] days; *p* = 0.017). Patients who developed SSI had longer total hospital stays (22 [IQR, 9–82] vs. 9 [IQR, 7–15] days; *p* = 0.014) and postoperative hospital stays (13 [IQR, 4–73] vs. 4 [IQR, 3–9] days; *p* = 0.021). Baseline characteristics of the study cohort and comparisons according to SSI status are presented in Table 1.

At admission, platelet count was the only laboratory parameter that differed significantly between the groups, with lower values observed in patients who subsequently developed SSI (176 [IQR, 162–233] vs. 226 [IQR, 192–293] × 10^9^/L, *p* = 0.029). Admission hemoglobin, neutrophil count, monocyte count, lymphocyte count, and the remaining inflammatory biomarkers showed no significant between-group differences. Likewise, preoperative and postoperative day 0 hematological parameters and inflammatory biomarkers were comparable between patients with and without SSI (all *p* > 0.05). Laboratory findings are summarized in Table 2.

In addition to the between-group comparisons reported above, univariable Cox proportional hazards regression analyses were performed to evaluate factors associated with the development of SSI (Table 3). Staged treatment was associated with a significantly increased hazard of SSI compared with direct definitive fixation (hazard ratio [HR] 4.14, 95% CI 1.31–13.0; *p* = 0.015). Each additional day between injury and definitive surgery was associated with a 16% increase in the hazard of SSI (HR 1.16, 95% CI 1.02–1.32; *p* = 0.023). Higher admission platelet count was associated with a lower hazard of SSI, with each 50 × 10^9^/L increase corresponding to an HR of 0.61 (95% CI, 0.38–0.97; *p* = 0.038), while admission SIRI was also associated with SSI in the univariable analysis (HR 1.03, 95% CI 1.00–1.05; *p* = 0.048).

In the multivariable Firth penalized logistic regression analysis, none of the included variables demonstrated a statistically significant independent association with SSI (Table 4). Staged treatment showed an adjusted OR of 4.01 (95% CI, 0.81–22.58; *p* = 0.088), while the adjusted OR for each additional day from injury to definitive surgery was 1.11 (95% CI, 0.90–1.33; *p* = 0.301). Age (per 10-year increase), male sex, and ISS (per 5-point increase) were also not significantly associated with SSI (all *p* > 0.05).

Exploratory ROC analyses showed limited discriminatory performance of the preoperative laboratory parameters and inflammatory biomarkers for SSI. None of the evaluated parameters demonstrated an AUC with a 95% CI excluding 0.50. Preoperative hemoglobin and monocyte count showed the highest AUCs (both AUC = 0.597), while preoperative SIRI demonstrated essentially no discriminatory ability (AUC = 0.505, 95% CI 0.335–0.674). Overall, the ROC findings did not support clinically meaningful discrimination of SSI by the evaluated preoperative biomarkers. Exploratory cutoff values with corresponding sensitivity and specificity estimates are presented in Supplementary Table S1.

Temporal changes in perioperative laboratory parameters were further evaluated using linear mixed-effects models. None of the evaluated parameters demonstrated a statistically significant time × SSI interaction after Benjamini–Hochberg adjustment for multiple comparisons. Platelet count showed the lowest unadjusted interaction *p* value (*p* = 0.050), but this was not statistically significant after adjustment (adjusted *p* = 0.908).

## 4. Discussion

The present study evaluated the association between routinely available perioperative inflammatory biomarkers and postoperative surgical site infection following pelvic and acetabular fracture surgery. Overall, most inflammatory indices were not associated with subsequent SSI, regardless of whether they were measured at admission, immediately before definitive surgery, or on postoperative day 0. Among the evaluated inflammatory biomarkers, only admission SIRI demonstrated a modest association with SSI in the univariable Cox regression analysis. Lower admission platelet counts were also associated with SSI. In univariable analyses, staged management and a longer interval to definitive fixation were also associated with SSI; however, these associations were attenuated and no longer statistically significant after adjustment in the Firth-penalized logistic regression model. These findings suggest that routinely available inflammatory biomarkers alone may have limited value for perioperative SSI risk assessment in this heterogeneous trauma population and underscore the importance of considering the broader clinical context when interpreting univariable associations.

The limited utility of most inflammatory indices observed in the present study is not entirely unexpected when the existing literature is considered. Although elevated inflammatory markers have been associated with postoperative complications in several orthopedic settings, the reported findings have been far from consistent. Most of the available evidence originates from geriatric hip fracture, arthroplasty, or spine surgery populations, in which inflammatory biomarkers such as NLR, PLR, SII, and MLR have been linked to outcomes including mortality, pneumonia, acute kidney injury, or surgical site infection [9,11,12,13,14,16]. However, other studies have failed to demonstrate similar associations for several of these indices, even within comparable orthopedic populations [17,18,19]. Such variability suggests that the prognostic performance of inflammatory biomarkers is likely influenced by differences in patient characteristics, injury patterns, clinical endpoints, and study design rather than representing universally applicable predictors across orthopedic trauma populations.

Among the evaluated inflammatory biomarkers, admission SIRI was the only index that showed an association with SSI in the univariable Cox regression analysis. This finding should be interpreted with caution, as the association was modest and was not supported by the direct between-group comparisons. SIRI incorporates neutrophil, monocyte, and lymphocyte counts and has been proposed as a marker reflecting systemic inflammatory activity. Previous studies have reported associations between elevated preoperative SIRI and adverse postoperative outcomes, including acute kidney injury after joint arthroplasty, postoperative infectious complications following spinal surgery, and mortality after hip fracture [9,14,20]. Whether this broader inflammatory profile offers additional prognostic value in patients with pelvic and acetabular fractures remains uncertain. Given the limited number of SSI events and the isolated nature of this finding, this association should be regarded as exploratory rather than as evidence of an independent relationship. These findings therefore require confirmation in larger prospective studies.

An additional finding of the present study was the association between lower admission platelet counts and subsequent SSI. Although platelet-based inflammatory indices, including PLR and SII, were not associated with infection, platelet count itself was associated with SSI. The available orthopedic literature has primarily focused on postoperative platelet-related changes or platelet-containing inflammatory indices rather than admission platelet counts, making direct comparisons with our findings difficult. The biological explanation for the observed association therefore remains uncertain. Given the limited number of SSI events in our cohort, this finding should be interpreted cautiously and considered hypothesis-generating until confirmed in larger studies.

In univariable analyses, staged management and a longer interval between injury and definitive fixation were associated with SSI. However, neither variable remained statistically significant in the multivariable Firth penalized logistic regression model after adjustment for age, sex, and injury severity. Staged treatment and delayed definitive fixation may reflect the broader clinical complexity of pelvic and acetabular trauma, as these management strategies are often influenced by injury severity, physiological status, and the conditions surrounding definitive fixation. Accordingly, the associations observed in the univariable analyses should not be interpreted as evidence that these treatment-related factors independently increase the risk of SSI. Previous studies have identified several clinical and injury-related factors, including severe associated injuries, obesity, Morel-Lavallée lesions, prolonged operative duration, and intensive care unit admission, as potential determinants of SSI after pelvic and acetabular fracture surgery [3,4,6,7,8]. Our findings therefore emphasize the need to consider treatment-related variables within the overall clinical context rather than as isolated risk factors. Hospital length of stay was not interpreted as a potential risk factor because prolonged hospitalization may represent a consequence rather than a cause of postoperative SSI.

This study has several strengths. To our knowledge, it is among the few studies to comprehensively evaluate routinely available inflammatory biomarkers at multiple perioperative time points in patients undergoing surgery for pelvic and acetabular fractures. In addition, SSI was defined according to standardized criteria, and inflammatory biomarkers were evaluated at three clinically relevant perioperative time points. Nevertheless, several limitations should be acknowledged. First, the retrospective single-center design may limit the generalizability of the findings. In addition, a substantial proportion of the initially screened patients were excluded based on the predefined eligibility criteria. Although most exclusions were inherent to the definition of the target study population, including nonoperative management, death before definitive fixation, and age < 18 years, the exclusion of patients with unavailable admission laboratory data and those with less than 12 months of follow-up may have introduced selection bias. Patients referred from other institutions without admission laboratory measurements may have differed in injury severity or clinical course from those presenting directly to our center, while insufficient follow-up may have resulted in incomplete ascertainment of postoperative SSI. Consequently, the analyzed cohort may not fully represent the overall population undergoing operative treatment for pelvic and acetabular fractures, and the observed associations between admission biomarkers and SSI should be interpreted with this potential selection bias in mind. Second, the relatively small number of SSI events limited the number of covariates that could be included in the multivariable model and resulted in wide confidence intervals around several adjusted estimates. Although Firth penalized logistic regression was used to reduce small-sample bias and improve estimate stability, the adjusted findings should therefore be interpreted cautiously. Furthermore, multiple laboratory parameters and inflammatory indices were evaluated, increasing the possibility of chance findings due to multiple testing. Accordingly, isolated statistically significant findings, particularly those from univariable analyses, should be considered exploratory and require external validation. Finally, although inflammatory biomarkers were evaluated at three clinically relevant time points, changes beyond the immediate postoperative period were not analyzed because of incomplete laboratory follow-up. In addition, data regarding perioperative blood transfusion and standardized assessment of soft-tissue injury severity were not consistently available in the retrospective records and therefore could not be evaluated. Future multicenter studies with larger cohorts are needed to validate these findings and further clarify the role of inflammatory biomarkers in this challenging patient population.

## 5. Conclusions

In conclusion, routinely available perioperative inflammatory biomarkers demonstrated limited utility for identifying patients at risk of surgical site infection following pelvic and acetabular fracture surgery. Among the evaluated biomarkers, admission SIRI and lower admission platelet counts were associated with SSI in univariable analyses; however, these findings should be considered exploratory. Staged management and delayed definitive fixation were also associated with SSI in univariable analyses, but neither remained statistically significant after adjustment in the multivariable Firth penalized logistic regression model. These findings highlight the multifactorial nature of postoperative SSI and the importance of cautious interpretation of isolated associations in this heterogeneous trauma population. Further prospective multicenter studies with larger cohorts are warranted to validate these observations.

## Figures and Tables

**Table 1:** Baseline characteristics of the study cohort according to SSI status.

Variable	Overall (*n* = 104)	No SSI (*n* = 92)	SSI (*n* = 12)	*p* Value
Age, years	41 (28–53)	41 (27–52)	41 (28–53)	0.955
Male sex	75 (72.1)	64 (69.6)	11 (91.7)	0.172
Diabetes mellitus	16 (15.4)	12 (13.0)	4 (33.3)	0.087
Pelvic ring fracture	41 (39.4)	37 (40.2)	4 (33.3)	0.760
Acetabular fracture	63 (60.6)	55 (59.8)	8 (66.7)	0.760
Staged treatment	16 (15.4)	11 (12.0)	5 (41.7)	0.019
Time from injury to definitive surgery, days	5.0 (3.0–6.5)	5.0 (3.0–6.0)	6.5 (4.5–10.0)	0.017
Operative duration, min	180 (120–240)	180 (120–240)	180 (135–225)	0.930
ISS	4.0 (0.0–9.0)	4.0 (0.0–9.0)	4.0 (4.0–9.0)	0.883

Values are presented as median (interquartile range) or *n* (%). ISS, Injury Severity Score.

**Table 2:** Comparison of perioperative inflammatory biomarkers according to surgical site infection status.

Variable	No SSI (*n* = 92)	SSI (*n* = 12)	*p* Value
***Admission parameters***			
Hemoglobin, g/dL	11.95 (10.20–13.95)	11.50 (10.80–13.10)	0.680
Neutrophils, ×10^9^/L	9.8 (7.6–11.5)	8.7 (6.6–11.4)	0.401
Monocytes, ×10^9^/L	0.70 (0.55–0.96)	0.71 (0.60–1.00)	0.629
Lymphocytes, ×10^9^/L	1.68 (1.08–2.60)	1.35 (0.96–1.47)	0.098
Platelets, ×10^9^/L	226 (192–293)	176 (162–233)	0.029
NLR	6 (3–10)	6 (4–12)	0.473
PLR	149 (94–224)	140 (114–196)	0.703
SII	1316 (736–2572)	1329 (698–2145)	0.867
SIRI	4.3 (2.1–8.0)	4.4 (2.8–10.2)	0.442
***Preoperative parameters***			
Hemoglobin, g/dL	10.60 (9.90–13.05)	10.45 (9.95–10.90)	0.276
Neutrophils, ×10^9^/L	7.3 (5.5–9.7)	6.8 (5.0–9.4)	0.600
Monocytes, ×10^9^/L	0.63 (0.49–0.90)	0.73 (0.59–0.89)	0.278
Lymphocytes, ×10^9^/L	1.48 (1.16–1.91)	1.50 (1.21–1.85)	0.919
Platelets, ×10^9^/L	252 (195–329)	270 (202–324)	0.784
NLR	5.0 (3.2–7.3)	3.9 (3.4–7.0)	0.579
PLR	168 (116–257)	186 (130–255)	0.835
SII	1376 (668–2248)	1096 (954–1956)	0.811
SIRI	3.3 (2.0–5.3)	3.4 (2.2–4.8)	0.963
***Postoperative day 0 parameters***			
Hemoglobin, g/dL	9.90 (8.95–11.10)	10.00 (9.20–10.60)	0.654
Neutrophils, ×10^9^/L	10.2 (7.7–11.8)	11.1 (8.5–13.2)	0.331
Monocytes, ×10^9^/L	0.72 (0.51–0.87)	0.82 (0.70–0.98)	0.077
Lymphocytes, ×10^9^/L	1.20 (0.83–1.63)	1.11 (0.98–1.58)	0.780
Platelets, ×10^9^/L	247 (190–332)	286 (231–420)	0.205
NLR	9 (6–13)	9 (7–11)	0.996
PLR	213 (149–313)	279 (107–343)	0.907
SII	2200 (1268–3648)	2711 (1070–4627)	0.538
SIRI	5.9 (3.5–9.4)	6.3 (5.5–9.3)	0.396

Values are presented as median (interquartile range). NLR, neutrophil-to-lymphocyte ratio; PLR, platelet-to-lymphocyte ratio; SII, systemic immune-inflammation index; SIRI, systemic inflammation response index.

**Table 3:** Univariable Cox proportional hazards regression analysis for factors associated with surgical site infection.

Variable	HR	95% CI	*p* Value
***Clinical variables***			
Age (per year)	1.00	0.96–1.03	0.794
Male sex	4.58	0.59–35.5	0.143
Diabetes mellitus	2.82	0.85–9.36	0.091
Acetabular fracture (vs. pelvic ring fracture)	1.31	0.39–4.35	0.664
Staged treatment (vs. direct definitive fixation)	4.14	1.31–13.0	0.015
Time from injury to definitive surgery (per day)	1.16	1.02–1.32	0.023
Operative duration (per minute)	1.00	0.99–1.01	0.938
***Admission laboratory parameters***			
Hemoglobin (per g/dL)	0.93	0.73–1.20	0.582
Neutrophils (per ×10^9^/L)	0.99	0.86–1.13	0.831
Monocytes (per ×10^9^/L)	2.73	0.63–11.9	0.180
Lymphocytes (per ×10^9^/L)	0.50	0.24–1.08	0.078
Platelets (per 50 × 10^9^/L)	0.61	0.38–0.97	0.038
NLR	1.02	0.96–1.09	0.471
PLR	1.00	0.99–1.00	0.341
SII	1.00	1.00–1.00	0.507
SIRI	1.03	1.00–1.05	0.048

Laboratory variables represent admission measurements. CI, confidence interval; HR, hazard ratio; NLR, neutrophil-to-lymphocyte ratio; PLR, platelet-to-lymphocyte ratio; SII, systemic immune-inflammation index; SIRI, systemic inflammation response index.

**Table 4:** Multivariable Firth penalized logistic regression analysis for surgical site infection.

Variable	Adjusted OR	95% CI	*p* Value
Staged treatment (vs. direct definitive fixation)	4.01	0.81–22.58	0.088
Time from injury to definitive surgery (per day)	1.11	0.90–1.33	0.301
Age (per 10 years)	0.91	0.57–1.40	0.685
Male sex	3.50	0.72–35.33	0.132
ISS (per 5 points)	0.74	0.34–1.36	0.347

CI, confidence interval; ISS, Injury Severity Score; OR, odds ratio.

## Data Availability

The datasets generated and/or analyzed during the current study are not publicly available due to institutional and patient privacy restrictions but are available from the corresponding author on reasonable request.

## Supplementary Materials

The following supporting information can be downloaded at: https://www.mdpi.com/article/10.3390/jcm15186981/s1, Supplementary Table S1. Exploratory receiver operating characteristic analysis of preoperative laboratory parameters and inflammatory biomarkers for surgical site infection.
