# Supplementary material for: Perioperative Inflammatory Biomarkers and Surgical Site Infection After Pelvic and Acetabular Fracture Surgery: A Retrospective Cohort Study
Source: J Clin Med. 2026 Sep 9;15(18):6981. doi: 10.3390/jcm15186981 (PMC13607970; doi:10.3390/jcm15186981)
Supplement: Supplementary file 1 [file jcm-15-06981-s001.zip › jcm-4527688-supplementary.pdf]

Supplementary Table S1. Exploratory receiver operating characteristic analysis of preoperative laboratory parameters and inflammatory biomarkers for surgical site infection

**Variable**	**AUC**	**95% CI**	**Exploratory cutoff**	**Sensitivity**	**Specificity**	***p value***
Hemoglobin	0.597	0.473–0.722	11.050	1.000	0.424	0.124
Neutrophils	0.453	0.271–0.635	4.145	1.000	0.076	0.612
Monocytes	0.597	0.449–0.745	0.475	1.000	0.239	0.200
Lymphocytes	0.490	0.321–0.660	1.360	0.500	0.674	0.913
Platelets	0.525	0.344–0.706	262.000	0.667	0.533	0.787
NLR	0.450	0.278–0.622	2.869	0.917	0.174	0.570
PLR	0.519	0.344–0.694	126.255	0.833	0.326	0.831
SII	0.478	0.307–0.650	896.656	0.833	0.359	0.804
SIRI	0.505	0.335–0.674	3.369	0.583	0.565	0.958

AUC, area under the curve; CI, confidence interval; NLR, neutrophil-to-lymphocyte ratio; PLR, platelet-to-lymphocyte ratio; SII, systemic immune-inflammation index; SIRI, systemic inflammation response index. P values represent tests of discrimination against an AUC of 0.50. Cutoff values were determined using the maximum Youden index, with corresponding sensitivity and specificity estimates. These thresholds were derived and evaluated in the same dataset and, given the limited number of SSI events, should be considered exploratory and should not be interpreted as validated clinical cutoffs.
